# Perception and utilization of public health services in Southeast Nigeria: Implication for health care in communities with different degrees of urbanization

**DOI:** 10.1186/s12939-016-0294-z

**Published:** 2016-01-20

**Authors:** Nkechi G. Onyeneho, Uche V. Amazigo, Ngozi A. Njepuome, Obioma C. Nwaorgu, Joseph C. Okeibunor

**Affiliations:** Department of Sociology/Anthropology, University of Nigeria, Nsukka, Nigeria; Takemi Program in International Health, Department of Global Health and Population, Harvard T.H, Chan School of Public Health, Harvard University, Boston, MA USA; P.O.Box 3397, Main Post Office, Okpara Avenue, Enugu, Nigeria; P.O.Box 7117, Wuse, Abuja, Federal Capital Territory Nigeria; Department of Parasitology, Nnamdi Azikiwe University, Awka, Nigeria; Immunization, Vaccines and Emergencies, WHO Regional Office for Africa, Brazzaville, Congo

**Keywords:** Perception, utilization, health services, Nigeria

## Abstract

**Background:**

The relationship between people’s perception and utilization of public health serviceswas investigated.

**Methods:**

A survey of 840 households across selected urban, peri urban and rural communities, in the Southeast of Nigeria, was conducted using the mixed methods approach.

**Results:**

Of the nine (9) demographic variables, only the locality and status of the health system (strong or weak in terms of child immunization) was found to influence both the poor rating and utilization of public health services. Individuals from states with strong health system rated relatively higher and used public health services more (p < 0.001), than their counterparts from states with weak health care system. Similarly, those in the urban or peri-urban localities used public health services more (p = 0.013). The two perceptual variables significantly influence the rating and use of public health services. Those with a good perception of the quality of health service provided, rated and patronized them more (p < 0.001). Also, health centres that provide a high number of services enjoyed greater rating and patronage (p < 0.001 and p = 0.0524 respectively). The results of the structured questionnaire survey were confirmed by qualitative enquiry,based on in-depth interviews and focus group discussions.

**Conclusions:**

It will be necessary to create a more responsive atmosphere in the health facilities, with culturally-sensitive and friendly health workers, and provision of affordable drug to improve the perceptions of the primary health care system, for it to succeed in providing health services for all.

## Background

Child and maternal deaths have experienced a significant reduction, reflecting progress towards the health-linked Millennium Development Goals (MDG) globally [1]. Between 1990 and 2013, global under-five mortality declined from 12.7 million to 6.3 million and maternal mortality ratio dropped from 380 to 210 per 100,000 live births [2]. However, the proportion of child and maternal deaths recorded in sub-Saharan Africa increased [3]. In Nigeria, for instance, maternal mortality ratio increased from 545 to 575 deaths per 100,000 live births between 2008 and 2013 [4, 5], reflecting a worsening situation [4, 6]. Interventions have been designed to reduce maternal mortality and achieve the MDG target of 250 or less deaths per 100,000 live births in Nigeria. Maternal and child health continue to worsen, despite the government’s partnership with the private sector to promote improved access to quality maternal health services [6].

The current realities indicate that the huge global and national investments aimed at effective health care delivery have not yielded the desired results because of poor utilization and ultimately low access. According to Frost and Reich [7], availability of interventions and services does not translate automatically to access. Access implies effective and appropriate utilization of the interventions and services by the intended clientele. Poor access to health facilities and services was identified as a factor militating against efforts to address major health problems in Africa [8].

Gaps have also been revealed between need and actual access to some critical health services [4]. Sixty-one percent of mothers in Nigeria received antenatal care from a skilled provider. Of this proportion, only 51 % made at least four antenatal care visits during pregnancy. This gives a 49 % point drop in utilization between the first and fourth usage. In terms of child health, only 19 % of the children (aged 12–23 months at survey time) received all the basic vaccinations as shown in the vaccination cards. The third of diphtheria, pertussis and tetanus (DPT3) vaccine, used as proxy for vaccine utilization, recorded only 22.2 % use, whereas DPT1 was 26.7 %.

Perception has come up a prominent determinant of the utilization of health services. According to Roberts et al. [9], “…utilization is only partially a reflection of effective availability, as patients may choose not to use services, even if they are available”. The decision to use available health services depends on people’s perception of the services and affordability. People’s perceptions and judgment are often conditioned by assessing factors their traditions and culture consider important such as courtesy, responsiveness, attentiveness, and perceived competence of the health staff [10]. Perceptions are determined by the people’s level of satisfaction with the health service, as well as their assessment of the attitude of health workers, which often determines whether they would return in future. To achieve universal health for the people, it is imperative that all stakeholders understand the people’s perception of health service, to ensure successful interventions. This is critical to developing appropriate promotional messages and campaigns, aimed at creating demand for particular health interventions [11].

Social-psychologists argue that perception, in the context of health, is structured on the basis of variables like ‘risk perception’ (the degree to which one feels susceptible to certain health risk), ‘self-efficacy’ (confidence in one’s ability to take the necessary action) and ‘action-outcome expectancies’ (ones belief that the proposed action is contributory to the expected outcome) [12–18]. Others stressed that these variables tend to produce motivation to adhere to healthier behaviours (precautionary motivation) that may lead to an intention to carry out these behaviours [19, 20]. It has also been argued that health care utilization is a function of both need-related factors as well as supply-induced and thus strongly dependent on the structures of the health care system [21]. Anderson’s behavioural model captures these different domains aptly in its multilevel model that incorporates both individual predisposing factors and contextual or health provider determinants of health service use otherwise called enabling and need factors [22]. However, data on perceptions of health interventions and services have generally been collected quantitatively with comparatively low reliance on qualitative methods of inquiry [23–30]. This study adopted the mixed-method approach, in assessing the perceptions of people in Southeast Nigeria and how it influences their utilization of public health facilities and services (that is primary health care).

## Methods

### Study design

**Fig. 1:**
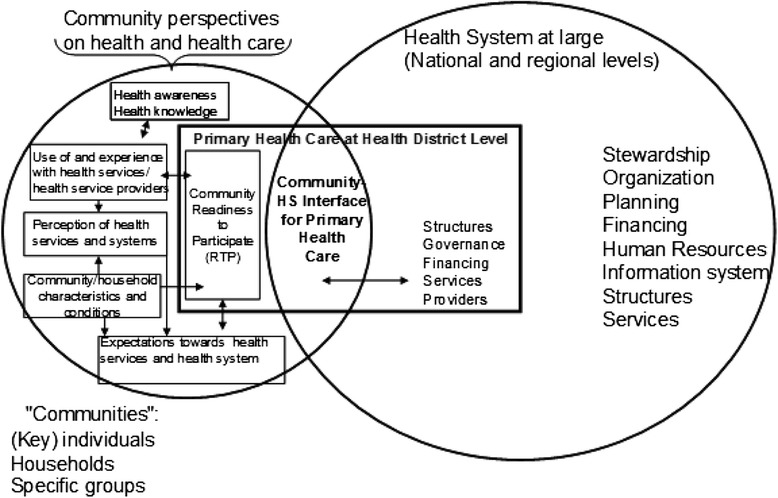
Framework for analysing community perceptions and perspectives on delivery of essential health services

This study was designed for description and analysis of community perceptions and expectations of district-based health care systems, in the South east geopolitical zone of Nigeria. Community perception of health and health care was assessed using a framework that recognizes interactions among a number of variables [31], such as the environmental and socio-demographic realities of the people, as well as their past experiences with the health system. See the Fig. 1. The framework accounts for past experiences with the PHC, in explaining the intermediate factors that influence perceptions and expectations from the PHC system. This multidisciplinary study combined two analytical designs, namely: 1) a cross-sectional survey and 2) a qualitative inquiry. The survey research was based on an interviewer-administered household survey instrument (structured questionnaire) while the qualitative inquiry was based on in-depth interviews and focus-group discussion methods. These two designs contributed to an in-depth, triangulated understanding of community perceptions and perspectives of health systems in Southeast Nigeria.

### Study area

**Fig. 2:**
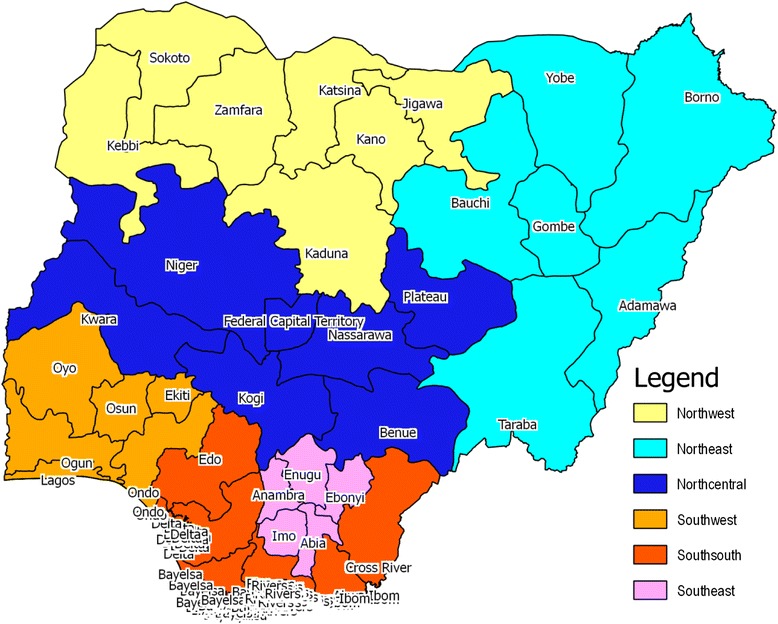
Map of Nigeria six geopolitical zones and showing the study sites (Imo & Enugu)

Figure 2 shows that the Nigerian political structure comprises of thirty-six states and the Federal Capital Territory distributed into six geopolitical zones (GPZs). The South East GPZ (comprising of five states) is predominantly Igbo, one of the three largest ethnic-sociocultural groups in Nigeria. Beyond being culturally homogenous, the zone had the highest average zonal immunization coverage (66.9 %) in 2008 and 88.9 % in 2013 [4].

States in the South East GPZ were grouped into two categories of “strong” (well performing) versus “weak” (less well performing) based on the zonal DPT3 coverage in the 2008 Nigeria Demographic and Health Survey [5]. Any state within the zone with less than the zonal average was classified as weak. Imo and Enugu States with 77.0 % and 50.0 % were randomly selected as strong and weak performing states, respectively.

### Population and sampling

The LGAs in each of the two selected states were classified and grouped into three clusters of rural, urban, and peri-urban. Three LGAs, one each from the urban, peri-urban, and rural segments of each sampled state, were randomly selected, for a total of six LGAs. From the list of communities in each sampled LGA, four communities were randomly selected. These formed the sampling clusters from which eligible respondents were drawn, following a two-stage sampling. The communities were grouped into two clusters of far (>5 km) and near (<5 km) to the LGA PHC center. Two communities were randomly selected from each cluster, yielding a total of twenty-four communities.

Using a 50 % assumed rate of health services awareness and a confidence interval of 95 % (estimated error margin of 3.5 %), a sample size of 770 ± 27 individuals was computed in the communities. The 50 % rate gives the highest probability of inclusion of the population elements in the study thereby enhancing the representativeness of the sample [32]. However, the sample size was rounded up to 840 households, taking into account a 5 % contingency rate. Approximately thirty-five household heads were interviewed in each of the twenty-four communities.

To select the households, a central location in each of the randomly selected communities was identified, which served as the starting point for data collection in the selected community. Two data collectors were assigned to cover each community cluster and the interviewers moved in opposite directions from the identified starting point in each community. Interviewers continued to turn right at every junction, until the desired number of respondents was attained. On occasions where the number required in any community was not reached, interviewers moved into an adjacent community to complete the number.

Community tradition heads were selected purposively for in-depth interview. Participants in the FGD were enlisted base on availability.

### Research instruments

Six different research instruments were employed in the study, each targeting different sources of information for investigating the research questions. These instruments included household survey instrument administered to 35 heads of households in each of the 24 communities, four different guides for in-depth interviews with community leaders and representatives of community based organization, community health volunteers, and health workers. Another set of instrument was the focus group discussion (FGD) guide. Twelve FGDs (three adult females and three adult males; three adolescent females and three adolescent males) with between eight and ten persons in each session were held in each health region. Each method had implications for different aspects of the study, unit of analysis and specific information about the needs of research questions.

### Data analysis

All quantitative data were computer processed with EPI Info version 6 and analyzed with SPSS version 19. Simple descriptive statistics were employed in characterizing the respondents. However, multivariate analyses were also conducted to detect the predictive powers of certain demographic characteristics of the respondents on their perceptions of health systems in South East Nigeria.

Qualitative data consisted of textual data, mainly transcripts from interviews and discussion data. All qualitative data were analyzed using Atlas.Ti. All interviews were tape-recorded and detailed notes were taken simultaneously, including verbal citations. Tape-recorded interviews were transcribed according to standard rules and translated into English. All textual data were entered into Atlas.Ti software, and coded according to an established code list. Citations, by code and memos, were analyzed according to emerging themes using the network visualization abilities of the Atlas.TI software for qualitative analysis.

### Ethical approval

The University of Nigeria Teaching Hospital Ethical Review Committee provided ethical approval for the conduct of the study.

## Results

### Socio-demographic characteristics

Table 1 summarizes the socio-demographic characteristics of the sample population. The mean age of the respondents was 52.15 years (52.15 ± 12.38SD), and over two-thirds (80.1 %) of them had lived in specific study sites for ≥11 years; thus, were able to relate to community events and health service activities with confidence. The respondents are predominantly males with only 25.8%females. This is however not strange, given the predominance of male headed households in African communities. More than two-thirds of the respondents were married (73.2 %) with a slightly higher proportion of the respondents in the rural communities (27.1 %) reporting widowhood. This could be due to the fact that the rural sample had a relatively higher proportion of older persons (46.8 % aged 55+ (n = 280)) compared to the urban and peri-urban sites. The main income generating activities include artisan (22.9 %), farming (20.6 %), followed by business (19.1 %), small scale trading (18.5 %) and paid employment (16.0 %). On the whole however, 86.8 % of the respondents were engaged in at least one form of income activity or the other. The respondents were predominantly Christians (96.7 %) and 81.4 % had received formal education. Radio was the commonest source of information (48.3 %), irrespective of the locality.

**Table 1:** Socio-Demographic Characteristics of Respondents by Locality (% in Parentheses)

Socio-demographic Characteristics	Locality			Total
	Urban	Peri urban	Rural	
Sex				
Male	202 (72.1)	226 (80.7)	195 (69.6)	623 (74.2)
Female	78 (27.9)	54 (19.3)	85 (30.4)	217 (25.8)
Age				
25–34	29 (10.4)	14 (5.0)	10 (3.6)	53 (6.3)
35–44	78 (27.9)	59 (21.1)	57 (20.4)	194 (23.1)
45–54	87 (31.1)	77 (27.5)	82 (29.3)	246 (29.3)
55–64	46 (16.4)	69 (24.6)	64 (22.9)	179 (21.3)
65+	40 (14.3)	61 (21.8)	67 (23.9)	168 (20.0)
*Mean*	*49.16*	*53.25*	*54.02*	*52.15*
*STD*	*12.03*	*12.16*	*12.44*	*12.38*
*Median*	*48.00*	*53.00*	*52.00*	*50.00*
*Minimum*	*26.00*	*30.00*	*30.00*	*26*
*Maximum*	*85.00*	*90.00*	*92.00*	*92*
Level of Education Attained				
None	44 (15.7)	36 (12.9)	75 (26.8)	155 (18.5)
Primary	81 (34.3)	121 (49.6)	110 (53.7)	312 (45.5)
Secondary	98 (41.5)	90 (36.9)	69 (33.7)	257 (37.5)
Post-Secondary	41 (17.4)	31 (12.7)	19 (9.3)	91 (13.3)
Vocational	15 (6.4)	2 (0.8)	7 (3.4)	24 (3.5)
Non Formal/Arabic	1 (0.4)	0 (0.0	0 (0.0)	1 (0.1)
Income Generating Activities				
None	41 (14.6)	38 (13.6)	32 (11.4)	111 (13.2)
Farming/Fishing/Livestock	32 (13.4)	47 (19.4)	71 (28.6)	150 (20.6)
Small Scale Trading	54 (22.6)	34 (14.0)	47 (19.0)	135 (18.5)
Paid Employment	37 (15.5)	46 (19.0)	34 (13.7)	117 (16.0)
Artisan	56 (23.4)	63 (26.0)	48 (19.4)	167 (22.9)
Business	56 (23.4)	41 (16.9)	42 (16.9)	139 (19.1)
Other	4 (1.7)	11 (4.5)	6 (2.4)	21 (2.9)
Marital Status				
Single	5 (1.8)	7 (2.5)	3 (1.1)	15 (1.8)
Married	215 (76.8)	212 (75.7)	188 (67.1)	615 (73.2)
Widowed	51 (18.2)	49 (17.5)	76 (27.1)	176 (21.0)
Divorced	6 (2.1)	10 (3.6)	8 (2.9)	24 (2.9)
Separated	3 (1.1)	2 (0.7)	5(1.8)	10 (1.2)
Religious Affiliation				
Christian	270 (96.4)	276 (98.6)	266 (95.0)	812 (96.7)
Muslim	4 (1.4)	2 (0.7)	2 (0.7)	8 (1.0)
African Traditional Religion	5 (1.8)	2 (0.7)	12 (4.3)	19 (2.3)
No Religion	1 (0.4)	0 (0.0)	0 (0.0)	1 (0.1)
Length of Stay				
<1 year	3 (1.1)	2 (0.7)	1 (0.4)	6 (0.7)
1–4 years	23 (8.2)	13 (4.6)	7 (2.5)	43 (5.1)
5–10 years	62 (22.1)	27 (9.6)	29 (10.4)	118 (14.0)
11–20 years	60 (21.4)	56 (20.0)	54 (19.3)	170 (20.2)
>20 years	132 (47.1)	182 (65.0)	189 (67.5)	503 (59.9)
Mean	*25.9*	*32.2*	*34.5*	*30.9*
STD	*20.2*	*19.5*	*20.4*	*20.3*
Median	*20.0*	*30.0*	*31.5*	*30.0*
Min	*1*	*1*	*1*	*1*
Max	*75*	*85*	*92*	*92*
Source of Health Information				
Radio	142 (50.7	151 (53.9)	113 (40.4)	406 (48.3)
Television	12 (4.3)	4 (1.4)	6 (2.1)	22 (2.6)
Pamphlets	1 (0.4)	0 (0.0)	1 (0.4)	2 (0.2)
Billboard	1 (0.4)	2 (0.7)	0 (0.0)	3 (0.4)
Community/town crier	39 (13.9)	63 (22.5)	90 (32.1)	192 (22.9)
Friends & Relatives	33 (11.8)	26 (9.3)	15 (5.4)	74 (8.8)
Churches & health centers	44 (15.7)	32 (11.4)	54 (19.3)	130 (15.5)
Don’t Know	8 (2.9)	2 (0.7)	1 (0.4)	11 (1.3)

### Perception and utilization of health care services

**Fig. 3:**
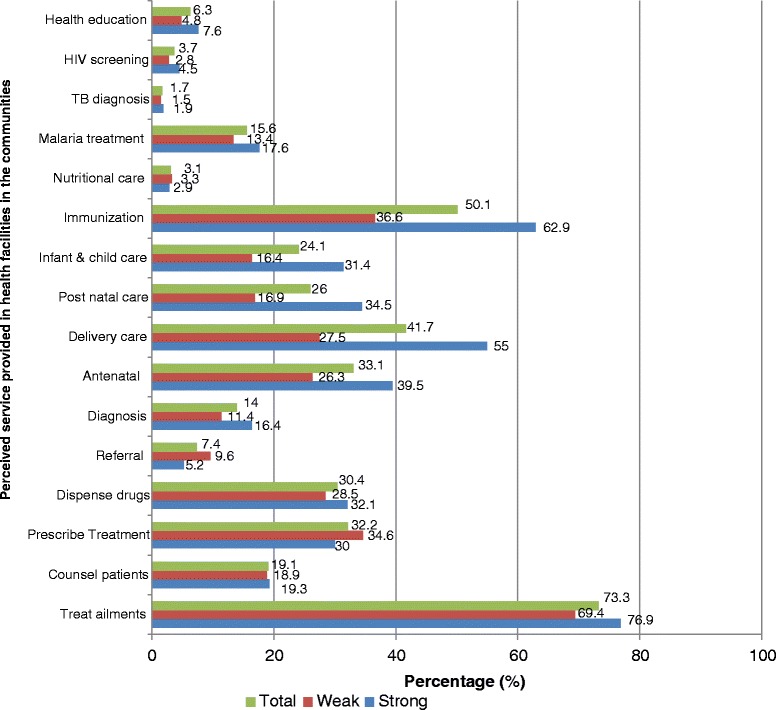
Perceived health services provided by government health facilities in the communities

Figure 3 revealed the emphasis on child and maternal care, irrespective of the health system’s strength. The inquiry on the people’s perception of health services available in the community health facilities was opened with a question of on the services they think were provided in the health facilities. It was a free listing process which allowed respondents to list as many services as they could unprompted, while the research assistants recorded against the relevant precoded options in the questions. Where any service mentioned was not covered in the list of options, the research assistants entered the under the category ‘other’. It was observed that care for the nutritional status patients, a major approach to maternal and child health care was very poorly represented in the services rendered in these facilities. Immunization and delivery services were also very poorly represented in the weak health system of the study. Generally, the perception of health services delivery was low, irrespective of the service or type of health system. Figure 2: Perceived health services provided by government health facilities in the communities.

Consequently, respondents in the study expressed poor perceptions of the general health services in the communities. More than half (50.4 %) the respondents rated health care services as bad, with the highest negative rating coming from rural dwellers (55.4 %), while peri-urban and urban respondents had an equally negative rating of 47.9 % each.

A majority of the respondents demanded that drugs be provided in the health facilities (74.9 %). The highest proportion of respondents (80 %), demanding that drugs be put in the health facilities, are from rural communities. Very high proportion (73.2 %, 61.4 % and 63.2 %) of urban, peri urban and rural respondents respectively were dissatisfied with the health care services, irrespective of locality.

Drugs were unavailable in about two-thirds (64 %) of the cases that sought health care 30 days prior to the survey. The implication is that the patients had to buy the prescribed drugs from medicine vendors, at prices with profit mark-ups. In 7.3 % of the cases, none of the prescribed drugs were available in the health facilities, with the rural communities being worst hit. About two-thirds (70.2 %) of the respondents had to get drugs from medicine shops.

Generally, health service delivery was rated low (≤50 % in many cases) in the community. This low rating cuts across the different social demographic backgrounds of the respondents. However, Table 2 shows that health care delivery in communities received a significantly higher rating *(p < 0.001),* among respondents from strong performing states. The results show that a significantly higher proportion of respondents from areas where health facilities are perceived to render more services, gave higher rating than their counterparts *(p < 0.001).*

**Table 2:** Demographic and perceptual correlates of health service rating and use in the communities

Characteristics			Rating of Services of Health Facility		Utilization of public health service	
Factors	Category	Number	% Good Rating	*χ* ^2^ Statistic; *p* value	Use of health services	*χ* ^2^ Statistic; *p* value
State	Strong	420	63.1	*χ* ^2^ = 145.84; *P* < 0.001	50.5	37.508
	Weak	420	21.9		29.8	<0.001
Proximity	Near	420	44.8	*χ* ^2^ = 2.01; *P* = 0.366	40.7	1.523
	Far	420	40.2		32.1	0.138
Locality	Urban	280	43.6	*χ* ^2^ = 5.24; *P* = 0.263	33.6	8.751
	Peri urban	280	44.6		45.7	0.013
	Rural	280	39.3		41.1	
Sex	Male	623	41.3	*χ* ^2^ = 5.69; *P* = 0.058	40.4	0.110
	Female	217	46.1		39.2	0.402
Education	Formal	685	43.4	*χ* ^2^ = 1.53; *P* = 0.466	40.4	0.157
	Non formal	155	38.7		38.7	0.381
Engage in Income Activity	Yes	729	41.4	*χ* ^2^ = 3.29; *P* = 0.193	39.9	0.093
	No	111	49.5		41.4	0.418
Length of Stay in the area (in years)	<1	6	50.0	*χ* ^2^ = 6.24; *P* = 0.621	16.7	
	1–4	43	51.2		39.5	
	5–10	118	45.8		41.5	1.735
	11–20	170	36.5		38.5	0.784
	>20	503	42.9		40.7	
Age (in years)	Young (<50)	368	43.2	*χ* ^2^ = 0.22; *P* = 0.897	38.8	0.766
	Old (50+)	472	41.9		41.8	0.211
Source of Health Information	Radio	406	45.4	*χ* ^2^ = 17.41; *P* = 0.235	40.6	
	Television	22	54.5		50.0	
	Pamphlet	2	50.0		50.0	
	Billboard	3	0.0		33.3	11.130
	Town crier	192	43.2		45.8	0.133
	Friend/Relatives	74	35.1		32.4	
	Church/HF	130	34.6		35.4	
	Don’t know	11	54.5		9.1	
Number of Services in Health Centre	Low (0–3)	426	34.0	*χ* ^2^ = 33.97; *P* < 0.001	37.3	2.822
	High (4–14)	390	54.1		43.0	0.054
Perception of health service	None existent	463	0.0	*χ* ^2=^789.18; *P* < 0.001	31.9	29.419
	Poor	53	9.5		47.2	<0.001
	Good	324	90.5		50.8	

In an interview, a patient summarized her frustration with poor services as follows:

*…if I had gone to a private hospital, I would have paid more money, but would have saved my time. I would be paid attention because the workers know that I’m bringing in money. They would be here on time to do their jobs but here, it is not like that. It is only poverty that will bring me here again…*

The respondents also differed in their perception of the number of services provided in health centres. For instance, more of the respondents (41.0 %) from areas where health centres are perceived to deliver more services, approved of the level of community involvement in health service delivery *(p < 0.001).* Despite variations by locality, the results show that more of the respondents (49.0 %) from strong performing states were satisfied with health care delivery. Compared to their urban counterparts, respondents in peri-urban (38.6 %) and rural (32.6 %) LGAs, were satisfied with health care delivery in their communities. During the exit interview, a patient had this to say, *“an injection that I should have taken at 6 am, was given to me at 11:45 because of their lateness, that is what I mean”.*

The respondents seemed to have matched their perceptions with the use of government health facilities in their communities. About half (50.2 %) of the respondents indicated that they patronize health centres, about forty percent (40.4 %) mentioned patent medicine vendor, while the remaining 10 % use other unorthodox means, for their health requirements. The peri urban area had the highest number of health centre users, while the least was from the urban area with 50.0 and 26.4 %, respectively.

Health centres and patent medicine vendors were very popular for the management of different health problems, irrespective of location. For instance, 57.1 % of the 840 respondents cited patent medicine vendors for the management of aches and pains while 40.1 % cited health centres. The health centres were popular for health problems like difficulty in moving, difficulty in engaging in vigorous activities, difficulty in caring for oneself, and difficulty in recognizing people. However, health centres were less popular in rural areas than in the urban or peri urban areas.

Participants in the qualitative inquiry stated lack of responsive services in government health centres as the reason for their preference of patent medicine stores. Respondents in the qualitative study also complained of lack of drugs and the poor attitude of health centre workers. The statement below illustrates this point.

*…It is heart breaking that when a sick person gets to the health centre, he or she will not get the drugs he needs.***[Participant: FGD, Adult Male in Imo, Peri Urban]**

*…the attitude of the health workers does not help matters. They treat us with no respect. In many cases, they respond to you based on their estimation of your social standing or wealth. The poor are ignored for the rich and even where they attend to you, they are very rude.***[Participant: FGD, Adult Female in Enugu, Rural]**

About a third (39.6 %) of the respondents had just one health care facility to visit. More of the urban respondents sought health care from private hospitals/clinics.

*When they are really sick, they look out for hospitals with qualified medical doctors… and other specialist medical personnel, whom they will not find at the primary health centres….***[Respondent: IDI; Health Worker in Owerri, Imo State]**

In the weak performing state however, a community leader in Ezeagu said,

*…because we don’t have a good hospital here, when people are sick they go to the bush and start looking for leaves and roots to boil and drink or they will go to the chemist to buy drugs that may be fake or just pray for the sickness to go away in God’s time…*

Table 2 shows low rates of use across different categories of the socio-demographic realities, with the exception of their state of origin and perceptions of health services. Respondents from Imo state, who were classified as having a strong health system, tended to use government health facilities more than their counterparts from Enugu State (*χ*2 = 37.508; p < 0.001). Similarly, those who perceived government health facilities to deliver a higher number of services tended to use the facilities more than their counterparts (p = 0.054). Also, individuals with a generally good perception of government health systems and services greatly used the facilities (p < 0.001).

Further analysis of the data was conducted using a logistic regression of the rating and utilization of government health facilities, using the socio-demographic characteristics of the respondents, as well as their perception of the health systems and services, as independent variables. Each independent variable in the regression models was converted into a dummy variable where the presence of a desired category was recorded as ‘1’ and the presence of an alternative recorded as ‘0’. The categories with higher scores were entered as the substantive and recorded as ‘1’ while the other categories were coded as ‘0’. For instance, the results in Table 2 shows that more peri-urban residents rated the health services as good and patronized them. Hence, the peri urban category was coded as ‘1’, while the rural and urban categories were coded as ‘0’. The results are as shown in Table 3.

**Table 3:** Regression of Respondents’ socio-demographic characteristics and perception of health service on rating and utilization of health facilities in the communities

Socio-demographic and perceptual characteristics	Rating of services in health facility		Utilization of health facility	
	B coefficient	*P* value	B coefficient	*P* value
Age (50 years +)	-0.570	0.142	0.180	0.240
Education (Formal)	0.347	0.502	0.005	0.978
Residence (peri urban)	0.905	0.013	0.442	0.005
Sex (male)	0.167	0.702	0.081	0.629
Income (Engage in income generation)	-0.006	0.992	-0.072	0.740
Perception of health service (Good)	0.357	0.324	0.698	0.000
Number of services in health centre (High)	24.021	0.991	0.096	0.513
Constant	-3.390	0.000	-0.666	0.014

Table 3 shows that variables in the two models, collectively influence the rating and utilization of health systems significantly (p < 0.001 and p = 0.014, respectively). However, only the place of residence (urban, peri urban or rural) contributed significantly to the capabilities of the regression models, in explaining the rating or utilization of health facilities in the communities. It shows that a unit increase in urbanization will lead to a 1.3 % point increase in the rating of the health facilities and 5 % increase in utilization. Similarly, a unit increase in the perception will lead to a 69.8 % point increase in health facilities utilization.

## Discussion

The general perception of health facilities service was poor across all the study areas (urban, peri-urban and rural). There was a general dissatisfaction with health care delivery based on the high cost of services in the rural and urban areas while the peri-urban area reported satisfaction with service delivery. The common health problems in all the study districts were malaria and fever. The signs of good health were based on physical activities such as the ability to work and move around. This poor understanding of the definition of health could be attributed to their level of education and exposure. The respondents viewed staying healthy as a product of healthy feeding, hygiene, sanitation and regular check-ups. All of which are in line with standards of best health practices.

The results of this study have not only corroborated previous studies on the impact of perception on health behavior, it also revealed significant association between the perceptions people hold of the quality and quantity of health services available in their various community health facilities and the use of such health facilities for any services. The people perceived community health facilities useful in the treatment of ailments, and prescription of drugs, but were grossly lacking in most preventive services, such as maternal and child health care among others.

The people often resorted to patent medicine vendors. Patent medicines are drugs that are allowed to be sold over the counter (OTC) that the Nigerian regulatory authorities, such as the Pharmaceutical Council of Nigeria (PCN), adjudge safe for unsupervised public use, as long as they are sold in their original manufacturer [32–36]. OTC drugs include common drugs like pain relieving tablets, antimalarials, cough syrups and so forth [36].

In many cases, community members who received care got prescriptions sourced from patent medicine stores. Nishtar [37] documented similar scenarios in his appraisal of health care delivery in African countries. He reported that in developing and underserved areas, a mixed health system characterized by government and private sector partnership, in which out-of-pocket payments and market provisions of services co-exists with publicly financed government health centres, is the norm.

Solutions to these health problems were sought mainly from government and private hospitals outside the study districts. However, ailments such as mental illness and convulsions were not taken to the hospitals. Patients with such ailments were mostly taken to traditional and spiritual healers, based on the cultural beliefs of the respondents. The household heads were mainly responsible for ensuring good health in the community, irrespective of district classification. This is not unusual in the African setting where men play significant roles in decision making.

Experiences of community engagement with the health system were facilitated by the avenues through which information reached them. The major source of information was the radio (48.3 %) followed by community announcement (22.9 %). There was no barrier to information on health and health service delivery; thus, suggesting good awareness on health issues. This was also confirmed by community involvement and participation in health issues, irrespective of the districts.

A comparative analysis of how information source influenced perceptions, revealed no difference with respect to perceptions on services of health facilities in the communities; community involvement and satisfaction with the way health care is provided in the communities. Similarly, the source of health information did not influence the perception of the adequacy of government contributions to community health. All of these point to a general low perception of the performance of the health system and government, in terms of health care delivery in the communities. However, with respect to the contributions of the communities, the source of health information affected perceptions significantly. In conclusion, the findings of the study revealed a poor community perception of health. The respondents in this study rated health systems poorly. Community expectations and responsiveness to health service delivery were not met. Health care delivery was hindered by lack of health facilities in some communities, shortage of commodities, and inaccessibility especially during the rainy season, as well as lack of basic amenities in the communities. Communities (urban, peri urban and rural) expressed disappointment for the grossly inadequate health care services available to them, characterized by acute shortage of drugs, poor attitude of health workers and lack of health insurance.

## Conclusions

There is widespread poor community perception of health irrespective of the tyoe of community. Community expectations of the heakth service seem largely unmet in an apparent weak healthsystem. A number of constraints to the functionality of the health system were highlighted to include lack of health facilities in some communities, shortage of commodities, and inaccessibility especially during the rainy season, as well as lack of basic amenities in the communities.
